# Supplementary material for: Trust and experiences of National Health Service healthcare do not fully explain demographic disparities in coronavirus vaccination uptake in the UK: a cross-sectional study
Source: BMJ Open. 2022 Mar 18;12(3):e053827. doi: 10.1136/bmjopen-2021-053827 (PMC8935008; doi:10.1136/bmjopen-2021-053827)
Supplement: Author's manuscript [file bmjopen-2021-053827.draft_revisions.pdf]

BMJ Open is committed to open peer review. As part of this commitment we make the peer review history of every article we publish publicly available.

When an article is published we post the peer reviewers' comments and the authors' responses online. We also post the versions of the paper that were used during peer review. These are the versions that the peer review comments apply to.

The versions of the paper that follow are the versions that were submitted during the peer review process. They are not the versions of record or the final published versions. They should not be cited or distributed as the published version of this manuscript.

BMJ Open is an open access journal and the full, final, typeset and author-corrected version of record of the manuscript is available on our site with no access controls, subscription charges or pay-per-view fees (http://bmjopen.bmj.com).

If you have any questions on BMJ Open's open peer review process please email info.bmjopen@bmj.com

BMJ Open

Trust and experiences of NHS healthcare do not fully explain demographic disparities in coronavirus vaccination uptake in the UK

Journal:	*BMJ Open*
Manuscript ID	bmjopen-2021-053827
Article Type:	Original research
Date Submitted by the Author:	29-May-2021

Complete List of Authors:	Allington, Daniel; King's College London, Dept. of Digital Humanities<br>McAndrew, Siobhan; University of Bristol, Sociology, Politics, and International Studies<br>Duffy, Bobby; King's College London, Policy Institute<br>Moxham-Hall, Vivienne; King's College London, Policy Institute

Keywords:	Public health < INFECTIOUS DISEASES, COVID-19, PUBLIC HEALTH

SCHOLARONE™
Manuscripts

I, the Submitting Author has the right to grant and does grant on behalf of all authors of the Work (as defined in the below author licence), an exclusive licence and/or a non-exclusive licence for contributions from authors who are: i) UK Crown employees; ii) where BMJ has agreed a CC-BY licence shall apply, and/or iii) in accordance with the terms applicable for US Federal Government officers or employees acting as part of their official duties; on a worldwide, perpetual, irrevocable, royalty-free basis to BMJ Publishing Group Ltd ("BMJ") its licensees and where the relevant Journal is co-owned by BMJ to the co-owners of the Journal, to publish the Work in this journal and any other BMJ products and to exploit all rights, as set out in our licence.

The Submitting Author accepts and understands that any supply made under these terms is made by BMJ to the Submitting Author unless you are acting as an employee on behalf of your employer or a postgraduate student of an affiliated institution which is paying any applicable article publishing charge ("APC") for Open Access articles. Where the Submitting Author wishes to make the Work available on an Open Access basis (and intends to pay the relevant APC), the terms of reuse of such Open Access shall be governed by a Creative Commons licence – details of these licences and which Creative Commons licence will apply to this Work are set out in our licence referred to above.

Other than as permitted in any relevant BMJ Author's Self Archiving Policies, I confirm this Work has not been accepted for publication elsewhere, is not being considered for publication elsewhere and does not duplicate material already published. I confirm all authors consent to publication of this Work and authorise the granting of this licence.

For peer review only

Trust and experiences of NHS healthcare do not fully explain demographic disparities in coronavirus vaccination uptake in the UK

26/5/2021

Daniel Allington (Corresponding Author)

Department of Digital Humanities, King's College London, UK

Address: Department of Digital Humanities, King's College London, Strand, London WC2R 2LS

Email address: daniel.allington@kcl.ac.uk

Siobhan McAndrew

Sociology, Politics, and International Studies, University of Bristol, Bristol, UK

Bobby Duffy

Policy Institute, King's College London, London, UK

Vivienne Moxham-Hall

Policy Institute, King's College London, London, UK

Word count: 4269

For peer review only

Trust and experiences of NHS healthcare do not fully explain demographic disparities in coronavirus vaccination uptake in the UK

Daniel Allington, Siobhan McAndrew, Bobby Duffy, and Vivienne Moxham-Hall

26/5/2021

Abstract

Objectives

To test whether demographic variation in vaccine hesitancy can be explained by differences in trust and healthcare experiences.

Design

Cross-sectional study.

Setting

Data collected online by Ipsos MORI in April 2021.

Participants

Data collected from 4885 UK-resident adults, of whom 3223 had received the invitation to be vaccinated against the novel coronavirus. The remainder were excluded. 1629 included participants identified as female and 1594 as male. 234 identified as belonging to other than white ethnic groups while 2967 identified as belonging to white ethnic groups.

Primary and secondary outcome measures

Uptake of coronavirus vaccination.

Results

Membership of an other than white ethnic group (AOR = 0.53, 95% CI [0.35, 0.84], p = .005) and age (AOR = 1.61, 95% CI [1.39, 1.87], p < .001 for a 1 SD change from the mean) were the only statistically significant demographic predictors of vaccine uptake. After controls for NHS healthcare experiences and trust in government, scientists, and medical professionals, the effect associated with membership of an other than

For peer review only

white ethnic group appears more marginal (AOR = 0.61, 95% CI [0.38, 1.01], p = .046), while the effect associated with age remains virtually unchanged. Exploratory analysis suggests that NHS healthcare experiences mediate 24% (95% CI [8%, 100%], p = .024) of the association between ethnicity and uptake, while trust mediates 94% (95% CI [56%, 100%], p .001) of the association between NHS healthcare experiences and uptake.

Conclusions

Members of other than white ethnic groups report inferior NHS healthcare experiences, which may explain the lower trust that they report in government, scientists, and medical professionals. However, this does not fully explain the ethnic gap in coronavirus vaccination uptake.

Trial registration

The data collection and confirmatory analysis were pre-registered with the Center for Open Science (osf.io/56txk).

Article summary

Strengths and limitations of this study

- The sample was large, providing high statistical power, and was designed for representativeness of the UK adult population
- Standard measures of trust and healthcare experiences were used, providing confidence that these variables have been measured robustly
- However, it was not possible to obtain a probability sample, and there was limited representation within the sample both of people who did not take up the invitation to be vaccinated and of members of other than white ethnic groups

1 Introduction

The problem of vaccine hesitancy, defined as ‘delay in acceptance or refusal of vaccination despite availability of vaccination services’ [1], has been recognised as a major global health problem for several years [2,3]. However, it has gained particular importance during the COVID-19 public health emergency: early in the pandemic, it was estimated that as much as 82% of a population might need vaccination or natural immunity against the disease before herd immunity could be reached [4], but one survey indicated that less than 72% of the British population might be willing to accept vaccination against SARS-CoV-2, the novel coronavirus which causes COVID-19 [5]. Multiple surveys found demographic disparities in British respondents’ expressed likelihood of vaccine uptake, with younger, less educated respondents, women, members of lower income households, and members of other than white ethnic groups all tending to express a lower probability of accepting the offer of vaccination [6–8]. The finding with regard to ethnicity has been of particular concern, given higher fatality rates for COVID-19 among members of minority ethnic groups

For peer review only

[9]. Actual uptake has been far higher than these surveys would have predicted, with one longitudinal study finding that 99% of those who had stated in December 2020 that they would accept vaccination, and 86% of those who had stated at the same point that they would not or were unsure, stated in February 2021 either that they would accept vaccination or that they had already done so [10]. However, despite this encouraging trend, demographic disparities appeared to persist [11], albeit at a reduced level, with youth and membership of other than white ethnic groups now being the strongest predictors of coronavirus vaccine hesitancy in the UK [10], such that by 13 January 2021, 42.5% of white people aged over 80 but not resident in care homes, but only 20.5% of black people in the same category, had been vaccinated [12]. The potential health consequences of incomplete vaccination coverage were recently highlighted by the official claim that the majority of people hospitalised as a result of a COVID-19 outbreak in the English town of Bolton were unvaccinated yet eligible to be vaccinated [13].

The possibility that lower trust in government, scientists, and the medical system might lead to reduced coronavirus vaccine uptake among members of minority ethnic communities had already been noted before survey data of this nature had been collected [14]. Indeed, many studies have found a relationship between hesitancy with regard to other vaccinations and mistrust in medicine and other relevant institutions, both on an individual level [15–18] and on a national level [19]. Evidence now exists to suggest that coronavirus vaccine hesitancy among African Americans may be driven by mistrust in the medical establishment and by perceptions of racism in the political system [20]. Moreover, some experts have attributed heightened levels both of coronavirus misinformation and of coronavirus vaccine hesitancy among members of some minority ethnic communities in the UK to mistrust which they argue is in turn driven by experiences of racism, discrimination, and exclusion [21]. This could be related to a UK study's finding that trust in scientists, medical professionals, and (to a much lesser extent) government is predictive of coronavirus vaccine hesitancy [8]. The same study found that ethnic disparities were reduced but did not disappear following controls for trust in these institutions, which may indicate the importance of other drivers. As members of minority ethnic groups report lower patient satisfaction and worse healthcare experiences in the UK [22], and, on an international level, appear to suffer from 'higher incidences of healthcare associated infections, dosing errors, [adverse drug events], and complications resulting from their care' [23], it would seem plausible that healthcare experiences (which were not assessed in the aforementioned study) may act as a driver of ethnic disparities in coronavirus vaccine uptake, alongside strategic and situational trust. Moreover, it would appear plausible that the range of mechanisms which can be assumed to generate the forms of exclusion associated with racism might also be associated with decreased trust, more negative experiences of healthcare, and higher rates of vaccine hesitancy among other groups, including women, young people, less highly educated people, and members of low income households. In this context, it is important to note that a separate UK study found that '[s]ocio-demographics do not explain vaccine hesitancy to any helpful degree' and that the major predictors of coronavirus vaccine hesitancy are 'excessive mistrust' and a lack of 'positive healthcare experiences' [5]. These findings are startling, and urgently require interrogation through replication.

This article therefore reports on a pre-registered study designed in order to test for demographic predictors of coronavirus vaccination uptake in the UK, both before and after controls for healthcare experiences and for trust in the principal institutions associated with the UK's coronavirus vaccination programme, i.e. government, scientists, and medical professionals [24]. As the UK's vaccination programme is delivered by National Health Service (NHS) bodies in England, Scotland, Wales, and Northern Ireland, healthcare experience items focused specifically on experiences of NHS healthcare. In order to provide the most robust

For peer review only - http://bmjopen.bmj.com/site/about/guidelines.xhtml

test possible, established instruments have been used to measure both trust and healthcare experiences, rather than the novel measures employed in the aforementioned study [5].

2 Hypotheses

Given the above observations regarding published findings of existing research, the following expectations were formed:

H1. Coronavirus vaccination uptake will be lower amongst female respondents
H2. Coronavirus vaccination uptake will be lower amongst younger respondents
H3. Coronavirus vaccination uptake will be lower amongst respondents of other than white ethnic groups
H4. Coronavirus vaccination uptake will be lower amongst less educated respondents
H5. Coronavirus vaccination uptake will be lower amongst respondents from lower-income households
H6. Coronavirus vaccination uptake will be positively associated with trust in government, medical professionals, and scientists
H7. Coronavirus vaccination uptake will be positively associated with positive experiences of healthcare

3 Methods

3.1 Study design

Trust was measured using Wellcome Global Health Monitor items Q11B, Q11E, Q14A, and Q15a [25]. Experiences of healthcare were measured using items adapted from the Scottish Government Inpatient Experience Survey [26]. Responses to individual questions were numerically recoded and averaged to provide a single measure of trust and a single measure of experience of healthcare. To facilitate analysis of individual trust and healthcare items, responses to these items were separately dichotomised for use in additional partial models, with the top two levels recoded as true and others recoded as false.

Education was operationalised as highest qualification received, which was dichotomised in order to distinguish degree-level (and equivalent) qualifications (including undergraduate and postgraduate degrees) from lower levels of qualification, including lack of all formal qualifications. Ethnic group was also dichotomised through coding as white or other than white, and household income was measured on an ordinal scale and dichotomised through coding as below median (for the sample) and median or above. In order to accommodate respondents who do not identify as male or female, gender was operationalised as a categorical variable with only two values: female and other than female. However, all participants included in the survey identified either as male or as female.

The dependent variable is a dichotomous variable representing the condition of having been vaccinated against the novel coronavirus (in the event that the respondent has been invited to be vaccinated) or of being about to attend an appointment to be vaccinated (in the event that a respondent was surveyed immediately after being invited to be vaccinated, and for that reason has not been vaccinated yet).

In calculating indices, missing values were ignored. Cases with missing data in relation to specific variables were excluded from all analyses featuring those variables.

For peer review only - http://bmjopen.bmj.com/site/about/guidelines.xhtml
For peer review only

3.2 Data collection

Although there is no longitudinal aspect to the study reported here, the data collection formed part of a longitudinal study in which each wave involves collection both from recontactees and from new participants. Respondents were sampled at random from an Ipsos MORI recruited panel, with pre-stratification in order to produce representativeness of the UK adult population on age, gender, geographical region, and working status. Where representativeness was not achieved, additional respondents were obtained by Ipsos MORI on a quota basis from panels maintained by other data providers. Data were collected from respondents who have not been offered vaccination against the novel coronavirus, but data on those respondents were excluded from this particular study, as they could not be used to test hypotheses about vaccine uptake.

Data collection was carried out online by Ipsos MORI. Fieldwork was launched on 01 April 2021 and completed on 16 April 2021. Informed consent was obtained from all participants. Demographic weights were calculated post-collection by Ipsos MORI on the basis of education and geographical region and of gender interlocked with age, NRS social grade, and working status. Weights were calculated before exclusion of cases where respondents had not yet been invited to receive coronavirus vaccination. Data were collected from a total of 4885 respondents, of whom 1662 were excluded due to not having received the invitation to be vaccinated. Numbers of included cases with missing values for each variable are listed in Table 3.1.

Table 3.1: Included cases with missing values

	Age	Gender	Degree	Other than white	Low income	Trust	NHS experiences	Uptake
Missing	0	0	0	22	305	8	124	46

3.3 Statistical analysis

The sample was treated as equivalent to a random sample for analytic purposes. Hypotheses 1-7 were tested through creation of binomial logit models with vaccine acceptance as the dependent variable. Of most relevant interest are a partial model using only demographic predictors, and the full model featuring demographic predictors and aggregate measures of trust and of experiences of NHS healthcare. Both of these models were pre-registered. To further probe the findings of the confirmatory analysis (see below), two further partial models were created without having been pre-registered. These included one featuring demographic predictors and aggregate measures of trust only and one featuring demographic predictors and aggregate measures of NHS healthcare only. In order to study the predictiveness of individual items, two further pre-registered models were created, one featuring demographic predictors and dichotomised responses to trust items as predictors, and the other featuring demographic variables and dichotomised responses to healthcare experience items as predictors. To further understand the unexpected findings of the confirmatory analyses, mediation analyses were conducted on an exploratory basis using non-parametric bootstrapping with 10000 repetitions. Coefficients for all confirmatory and exploratory analyses are reported both as estimates and as 95% confidence intervals.

3.4 Power analysis

Power analysis confirms that the sample was sufficiently large to detect even very small effects, at least in

For peer review only - http://bmjopen.bmj.com/site/about/guidelines.xhtml

the pre-registered models. Given a sample size of 2773 (i.e. the number of observations which were sufficiently complete to be fitted in the full model) and a cut-off of $p < .050$ (two-tailed), an association so weak as to reduce the probability of vaccine uptake only from 95% to 91% across the entire range of a predictor variable could be detected with approximately 98% power.

3.5 Patient and public involvement

No patient involved.

4 Results

4.1 Descriptive statistics and internal reliability

Table 4.1: Descriptive statistics: demographic variables

		Age		Gender		Degree		Ethnic group[a]		Household income[b]		
	n	M	SD	F.	M.	Yes	No	W.	O.	L.	M.	H.
**All invited**												
Raw	3223	52.00	14.64	51	49	39	61	93	7	43	19	38
Weighted		51.89	14.70	51	49	28	72	92	8	44	20	37
**Uptake: yes**												
Raw	2990	52.78	14.40	51	49	40	60	94	6	43	19	38
Weighted		52.70	14.48	51	49	28	72	93	7	44	19	37
**Uptake: no**												
Raw	187	44.10	13.83	50	50	34	66	85	15	42	23	35
Weighted		44.01	13.65	49	51	24	76	84	16	42	24	33

[a] White ethnic groups comprise white British, English, Welsh, Scottish, Northern Irish, Irish, Gypsy or Irish Traveller, and any other white background; other than white ethnic groups comprise all other ethnic categories used in the 2011 UK census, including mixed ethnicities

[b] Low household income = £24,999 or less; median household income (for included participants) = £25,000 - £34,999; high household income = £35,000 or more

Cronbach's alpha and Guttman's lambda 6 were used to assess internal reliability of indices. The reliability of items used to measure experiences of NHS healthcare was assessed as $\alpha = .88$, $\lambda_6 = .87$, which indicates a high level of internal reliability. The reliability of items used to measure trust was assessed as $\alpha = .69$, $\lambda_6 = .66$, which would be a suboptimal level of internal reliability for a conventional Likert scale but likely reflects real differences in levels of trust for different institutions (especially given that government trust may be acting at least partly as a proxy for political preference).

Table 4.2: Descriptive statistics: indices

		NHS exp.		Trust	
	**n**	**M**	**SD**	**M**	**SD**
**All invited**					
Raw	3223	4.06	0.71	3.09	0.52
Weighted		4.05	0.71	3.08	0.53
**Uptake: yes**					
Raw	2990	4.08	0.69	3.12	0.48
Weighted		4.08	0.69	3.12	0.48
**Uptake: no**					
Raw	187	3.70	0.92	2.64	0.77
Weighted		3.69	0.94	2.64	0.78

Table 4.3: Key variables: bivariate correlations

	**1**	**2**	**3**	**4**	**5**	**6**	**7**	**8**
1. Age		-.01	-.27	.11	-.09	.10	.06	.14
2. Female	-.01		-.04	.05	.05	.01	.05	.00
3. Other than white	-.27	-.04		-.01	.03	-.13	-.08	-.08
4. Low income	.11	.05	-.01		-.21	-.03	-.06	.00
5. Degree	-.09	.05	.03	-.21		.04	.04	.03
6. NHS experiences	.10	.01	-.13	-.03	.04		.47	.13
7. Trust	.06	.05	-.08	-.06	.04	.47		.22
8. Uptake	.14	.00	-.08	.00	.03	.13	.22	

Table 4.1 shows weighted and unweighted demographic descriptive statistics, first for the whole sample, and then for respondents who did and did not accept the invitation to be vaccinated against the novel coronavirus. Although respondents from households with income at or above the median for the sample were treated as a single group for modelling purposes, they are disaggregated here. Comparison with official statistics suggests slight representation of white ethnic groups, which accounted for 86% of the population at the 2011 census, but the sample was biased towards older members of the population as vaccination was offered in descending order of age, and other than white ethnic groups have a younger age profile in the UK [27].

The mean age for respondents who had been invited to be vaccinated yet not take up the invitation is just under nine years younger than the mean age for respondents who had been vaccinated. Percentages of male and female and respondents in each group are identical. Percentages with low (below median) household incomes for the sample are effectively identical, but the weighted percentage of respondents with high (above median) household incomes for the sample is 4 points lower among those who did not accept the invitation to be vaccinated (i.e. it was about 0.9 times as high). This suggests that the effect of income may be masked in the confirmatory analysis presented in this article, as dichotomisation by separating low income households from median and high income households was a pre-registered data transformation: a point to which we shall return below. The weighted percentage of respondents educated to degree level was 4 points lower among those who did not accept the invitation to be vaccinated (i.e. it was about 0.9 times as high). The weighted percentage of respondents belonging to other than white ethnic groups was 9 points higher among those who

For peer review only

did not accept the invitation to be vaccinated (i.e. it was about 2.3 times as high). This is not a large difference: by the time of data collection, 94% and 87% of invited members of other than white ethnic groups within the sample who had received the invitation to be vaccinated had taken it up, which implies that the great majority of members of all ethnic groups had done so. However, it is consistent with the observation, discussed above, that uptake rates have been lower amongst members of other than white ethnic groups.

Table 4.2 shows weighted and unweighted indices of trust and NHS healthcare experiences, first for the whole sample, and then for respondents who did and did not accept the invitation to be vaccinated against the novel coronavirus. Mean scores for members of the latter group were lower on both indices. Table 4.3 shows unweighted product-moment correlations between pairs of variables. There was a strong correlation between indices of trust and NHS healthcare experiences (r = .47), but NHS healthcare experiences were less strongly correlated with vaccine uptake (r = .13) than trust was (r = .22), while trust was less strongly correlated with membership of an other than white ethnic group (r = -.08) than NHS healthcare experiences were (r = -.13). Age was negatively correlated with membership of an other than white ethnic group (r = -.27) and with degree level education (r = -.09), but positively correlated with membership of a low income household (r = .11) and with vaccination uptake (r = .14).

For peer review only

Table 4.4: Partial models and full model

	AOR	2.5%	97.5%	p
**Demographics only**				
Age	1.61	1.39	1.87	<.001
Female	0.96	0.71	1.30	.808
Other than white	0.53	0.35	0.84	.005
Low income	1.02	0.75	1.40	.883
Degree	1.30	0.92	1.87	.140
**NHS experiences**				
Age	1.56	1.34	1.83	<.001
Female	0.98	0.71	1.34	.882
Other than white	0.65	0.41	1.04	.064
Low income	1.09	0.79	1.51	.591
Degree	1.29	0.91	1.87	.167
NHS experiences	1.46	1.27	1.67	<.001
**Trust**				
Age	1.57	1.34	1.85	<.001
Female	0.89	0.65	1.23	.490
Other than white	0.61	0.39	0.98	.037
Low income	1.12	0.81	1.56	.491
Degree	1.18	0.82	1.71	.380
Trust	2.06	1.80	2.36	<.001
**Full model**				
Age	1.57	1.33	1.86	<.001
Female	0.92	0.66	1.28	.615
Other than white	0.61	0.38	1.01	.046
Low income	1.16	0.83	1.63	.379
Degree	1.22	0.84	1.80	.297
NHS experiences	1.02	0.86	1.20	.820
Trust	2.02	1.72	2.37	<.001

For peer review only

Table 4.5: Individual trust and NHS healthcare experience items (dichotomised)

	AOR	2.5%	97.5%	p
**NHS experiences**				
Age	1.51	1.30	1.77	<.001
Female	0.91	0.67	1.24	.556
Other than white	0.64	0.41	1.01	.049
Low income	1.03	0.75	1.41	.848
Degree	1.24	0.88	1.79	.226
Respect	0.99	0.61	1.59	.967
Care	1.17	0.72	1.89	.513
Needs	1.45	0.96	2.18	.077
Trust staff	1.62	1.01	2.58	.045
Understand	1.37	0.89	2.11	.148
Spiritual	0.76	0.54	1.07	.113
**Trust**				
Age	1.52	1.30	1.78	<.001
Female	0.91	0.67	1.24	.552
Other than white	0.68	0.43	1.09	.097
Low income	1.12	0.82	1.54	.487
Degree	1.20	0.84	1.72	.322
Trust UK gov.	1.48	1.07	2.07	.021
Trust sci. uni.	1.77	1.12	2.77	.013
Trust sci. corp.	1.94	1.34	2.78	<.001
Trust medics	2.81	1.74	4.49	<.001

4.2 Confirmatory analysis

Table 4.4 presents adjusted odds ratios (with 95% confidence intervals) for dichotomous variables (female gender, membership of an other than white ethnic group, membership of a low income household, and education to undergraduate or postgraduate degree level) and for increases of 1 SD in numeric variables (i.e. age and the indices for trust and NHS healthcare experiences). Age (AOR = 1.61, 95% CI [1.39, 1.87], $p < .001$) and membership of an other than white ethnic group (AOR = 0.53, 95% CI [0.35, 0.84], $p = .005$) were the only statistically significant predictors in the purely demographic model. Thus, the null hypothesis can be rejected with regard to H2 and H3, but not with regard to H1, H4, and H5.

Controlling for indices of trust and of NHS healthcare experiences diminishes the effect associated with membership of an other than white ethnic group from AOR = 0.53, 95% CI [0.35, 0.84], $p = .005$ to AOR = 0.61, 95% CI [0.38, 1.01], $p = .046$. This suggests that some of the effect associated with ethnicity may be mediated by one or both of those additional variables. However, the same cannot be said for age. Indeed, the null hypothesis can only be rejected for H3 with regard to this model at $p < .050$, but the coefficient and p-value relevant to H2 are virtually unchanged relative to the demographic-only model. Moreover, while there was a significant effect associated with trust in the full model (AOR = 2.02, 95% CI [1.72, 2.37], $p < .001$), there was no effect associated with NHS healthcare experiences (AOR = 1.02, 95% CI [0.86, 1.2], $p = .820$).

For peer review only

For peer review only - http://bmjopen.bmj.com/site/about/guidelines.xhtml

Thus, the null hypothesis can be rejected with regard to H6, but not with regard to H7.

This finding would appear to be in contradiction of the claim that vaccine hesitancy was largely explained by trust alongside healthcare experiences, and that demographic predictors have little importance. Partial models excluding trust or healthcare experiences are also presented in Table 4.4. As the effect associated with NHS healthcare experiences in the partial model excluding trust is very highly significant (AOR = 1.46, 95% CI [1.27, 1.67], p < .001), albeit lower than the effect associated with trust in the partial model excluding NHS healthcare experiences (AOR = 2.06, 95% CI [1.8, 2.36], p < .001, AOR = 2.06, 95% CI [1.8, 2.36], p < .001), it seems plausible that the effect of NHS healthcare experiences on uptake may be mediated by trust: a possibility to which we shall return below.

Table 4.5 presents adjusted odds ratios (with 95% confidence intervals) for increases of 1 SD in age and for dichotomous variables, including dichotomised trust and NHS healthcare items as individual predictors. Examination of coefficients associated with those items facilitates assessment of their individual predictive contribution. All effects associated with trust items are statistically significant, especially those associated with trust in medical professionals (AOR = 2.81, 95% CI [1.74, 4.49], p < .001) and with scientists working in the private sector (AOR = 1.94, 95% CI [1.34, 2.78], p < .001). Thus, high trust in any of the institutions mentioned was associated with increased likelihood of uptake, even after controlling for level of trust in all the others, plus demographic variables. Interestingly, the only significant effect associated with an individual NHS healthcare experience item after controlling for all the other items and for demographic variables was that associated with having felt able to trust the staff (AOR = 1.62, 95% CI [1.01, 2.58], p = .045). In relation to these models, the null hypothesis is still rejected with regard to H6, and may be partially rejected with regard to H7.

4.3 Exploratory analysis

The above findings were suggestive of theoretically plausible mediated associations, i.e. that experiences of NHS healthcare might be indirectly associated with uptake via trust, that ethnicity might be indirectly associated with trust via experiences of NHS healthcare, and that ethnicity might therefore be indirectly associated with uptake via both of the aforementioned. For this reason, additional mediation analyses were carried out on an exploratory basis (i.e. without having been pre-registered). All of these analyses involved the same demographic controls used in the confirmatory analyses.

There were three findings. Firstly, the relationship between membership of an other than white ethnic group and trust appears to be 100% (95% CI [48%, 100%], p = .040) mediated by NHS healthcare experiences.[1] Secondly, the relationship between NHS healthcare experiences and uptake appears to be 94% (95% CI [56%, 100%], p .001) mediated via trust. Thirdly, the association between membership of an other than white ethnic group and uptake appears to be 24% (95% CI [8%, 100%], p = .024) mediated via NHS healthcare experiences (trust was not included in this model, given the second finding). This mediating effect may plausibly be taken to account for the reduction in the effect associated with ethnicity between the demographic-only model and the full confirmatory model.

While these findings are exploratory rather than confirmatory, they add weight to the above interpretation of the confirmatory findings. On the one hand, they suggest that positive experiences of NHS healthcare may be associated with higher vaccine uptake largely for the reason that they have a positive relationship with trust in relevant institutions, and that membership of an other than white ethnic group is associated with lower

For peer review only - http://bmjopen.bmj.com/site/about/guidelines.xhtml

trust in those same institutions for the reason that experiences of NHS healthcare are less positive among members of such groups. On the other hand, these findings also suggest that those intermediary relationships can only account for some of the association between ethnicity and vaccine uptake. Most of that association remains unexplained, as does the association between age and uptake.

5 Discussion

The finding that female gender, below degree-level education, and below median income do not predict uptake contradicts several studies which measured vaccine hesitancy before the UK vaccination campaign had got underway [6–8,10]. This discrepancy may be interpreted in terms of an attitude change potentially attributable to public health communication, word of mouth discussion of coronavirus vaccination itself, or many other sources (although it is also possible that members of certain groups simply under-estimated their own likelihood of accepting vaccination).

The finding that age and ethnicity do, however, predict uptake even after controls for trust and NHS healthcare experiences is in contradiction of the earlier finding that vaccine hesitancy is largely explained by mistrust and poor experiences of healthcare, to such an extent that demographic variables are not predictive once these variables are controlled for [5]. Non-replication of that finding might be accounted for by the timeframe (data collected after the vaccination programme had begun), outcome measure (actual uptake rather than expected uptake), predictor measures (those used here both for trust and for healthcare experiences were more standard), or analytic approach (pre-registered models rather than exploratory modelling). Age is here found to remain a powerful predictor of uptake even after those controls, and ethnicity also predicts uptake, albeit that this effect appears weaker after the same controls. This reduction in strength of association appears to be the result of mediation by NHS healthcare experiences, via trust. However, the mediation effect is small, appearing to account for about a quarter of the variation in uptake which is associated with ethnicity. To summarise, this study finds evidence that uptake of coronavirus vaccination is lower among members of other than white ethnic groups, that NHS healthcare experiences have a relationship with vaccine uptake that is mediated by trust, and that the lower trust in medics, scientists, and government among members of other than white ethnic groups is mediated by their worse experiences of NHS healthcare. However, it does not find evidence that worse healthcare experiences or lower trust can explain more than a small part of the ethnicity gap in coronavirus vaccine uptake.

This finding (or non-finding) suggests that lower rates of vaccination uptake among members of other than white ethnic groups are likely to have drivers other than or in addition to mistrust of medical, scientific, and other authorities and to negative experiences of healthcare. Experiences of healthcare explain little or no variation in uptake beyond that which is explained by trust, their association with uptake being almost entirely mediated by the latter. In some ways, this is encouraging: had the effect associated with ethnicity been found to be entirely explicable in terms of worse experiences of healthcare, then prospects of reducing it in the short term — and especially under pandemic conditions — would appear very bleak. However, the fact that so much variance remains unexplained is in itself a cause for concern, and highlights the urgent need for further research to test the mediating role of other variables. Possible candidate mediators involve sources of information, conspiracy beliefs about the novel coronavirus, and attitudes to vaccination in general [8]. Moreover, the finding that ethnic disparities in trust — a vital component in public health — could be so closely related to poorer reported experiences of NHS healthcare should serve as a reminder of the very real problem of ethnic disparities in healthcare experiences and outcomes, whether those disparities can be

For peer review only - http://bmjopen.bmj.com/site/about/guidelines.xhtml

assumed to have a population-level impact on coronavirus vaccine uptake or not.

6 Limitations of this study

This study relies on self-report data collected, and as such, it is dependent both on respondents' candour and on their recall. Also, while much effort has been made to produce a representative sample, it was not possible to obtain a probability sample. Moreover, as noted above, the focus on low income as a predictor (to which the analysts were committed by the pre-registration document) may have concealed the importance of high income as a predictor. Lastly, while the researchers were working with a relatively large sample, it included only 187 individuals who did not take up the invitation to be vaccinated, and only 234 members of other than white ethnic groups. Given that any effects associated with ethnicity must be disentangled from those associated with age, which in this context functions as a confound (both because age had a stronger association with uptake than ethnicity and because members of other than white ethnic groups were on average younger than members of white ethnic groups in the sample, as in the wider UK population), special caution must therefore be exercised in interpreting the results presented here. Further study of relationships such as those focused on in this study, for example looking for variables that might mediate the relationship between age and uptake, or that might explain the currently unexplained majority of the relationship between ethnicity and uptake, might therefore benefit either from an even larger sample or from deliberate over-sampling of individuals who have not taken up the invitation to be vaccinated, and perhaps also of members of specific demographic groups.

7 Technical note

The analysis presented here was carried out using R v 4.0.5 [28], with use of the following packages: psych v. 2.1.3 [29] for calculation of internal reliability of scales, Hmisc v. 4.5.0 [30] for calculation of weighted standard deviations, BBmisc v. 1.11 [31] for standardisation of numeric variables, mediation v. 4.5.0 [32] for mediation analysis, and WebPower v. 0.6 [33] for power analysis.

8 Contributorship statement

All four authors contributed to the design of the data collection instrument. Daniel Allington planned, pre-registered, and executed the analysis presented here, and wrote this article together with Siobhan McAndrew, who has overall leadership of the project within which this research took place.

9 Funding statement

This research was supported by the Economic and Social Research Council, ES/V 015494/1.

10 Ethics approval statement

King's College London ethics committee, MRA-20/21-21420.

For peer review only

11 Data sharing statement

Data will be made available following the conclusion of the project's period of funding.

12 Competing interests statement

The authors have no competing interests to report.

For peer review only

References

1
MacDonald NE, SAGE Working Group on Vaccine Hesitancy. Vaccine hesitancy: Definition, scope and determinants. *Vaccine* 2015;**33**:4161–4.
2
Dubé E, Gagnon D, Nickels E, *et al.* Mapping vaccine hesitancy—country-specific characteristics of a global phenomenon. *Vaccine* 2014;**32**:6649–54. doi:10.1016/j.vaccine.2014.09.039
3
WHO. Ten threats to global health in 2019. World health organisation. 2019.https://www.who.int/news-room/spotlight/ten-threats-to-global-health-in-2019
4
Sanche S, Lin Y, Xu C, *et al.* High contagiousness and rapid spread of Severe Acute Respiratory Syndrome Coronavirus 2. *Emerging Infectious Diseases* 2020;**26**:1470–7. doi:10.3201/eid2607.200282
5
Freeman D, Loe BS, Chadwick A, *et al.* COVID-19 vaccine hesitancy in the UK: The Oxford coronavirus explanations, attitudes, and narratives survey (OCEANS) II. *Psychological Medicine* 2020;1–5. doi:10.1017/S0033291720005188
6
Callaghan T, Moghtaderi A, Lueck JA, *et al.* Correlates and disparities of COVID-19 vaccine hesitancy. *Social Science & Medicine* 2021;**272**. doi:10.1016/j.socscimed.2020.113638
7
Allington D, McAndrew S, Moxham-Hall VL, *et al.* Media usage predicts intention to be vaccinated against SARS-CoV-2 in the US and the UK. *Vaccine* 2021;**39**:2595–603. doi:10.1016/j.vaccine.2021.02.054
8
Allington D, McAndrew S, Moxham-Hall V, *et al.* Coronavirus conspiracy suspicions, general vaccine attitudes, trust, and coronavirus information source as predictors of vaccine hesitancy among UK residents during the COVID-19 pandemic. *Psychological Medicine* 2021;1–7. doi:10.1017/S0033291721001434
9
ONS. *Updating ethnic contrasts in deaths involving the coronavirus (COVID-19), England and Wales: Deaths occurring 2 March to 28 July 2020*. Office for National Statistics 2020. https://www.ons.gov.uk/peoplepopulationandcommunity/birthsdeathsandmarriages/deaths/articles/updatingethniccontrastsindeathsinvolvingthecoronaviruscovid19englandandwales/deathsoccurring2marchto28july2020
10
Byrne T, Patel P, Shrotri M, *et al.* Trends, patterns and psychological influences on COVID-19 vaccination intention: Findings from a large prospective community cohort study in england and wales (virus watch). *medRxiv* Published Online First: 2021. doi:10.1101/2021.03.22.21254130
11
Office for National Statistics. Coronavirus and vaccine hesitancy, Great Britain: 13 january to 7 february 2021. 2021.https://www.ons.gov.uk/peoplepopulationandcommunity/healthandsocialcare/healthandwellbeing/bulletins/coronavirusandvaccinehesitancygreatbritain/13januaryto7february2021
12

For peer review only

O'Dowd A. Covid-19: Black people over 80 in England are half as likely as white people to have been vaccinated. *BMJ* 2021;**372**. doi:10.1136/bmj.n357
13
Elgot J. Hancock: Most Bolton Covid patients eligible for jab but haven't had it. *Guardian* Published Online First: 16 May 2021.https://www.theguardian.com/world/2021/may/16/hancock-defends-india-travel-ban-delay-as-covid-variant-cases-mount
14
Schaffer DeRoo S, Pudalov NJ, Fu LY. Planning for a COVID-19 vaccination program. *JAMA* 2020;**323**:2458–9. doi:10.1001/jama.2020.8711
15
Mills E, Jadad AR, Ross C, *et al.* Systematic review of qualitative studies exploring parental beliefs and attitudes toward childhood vaccination identifies common barriers to vaccination. *Journal of Clinical Epidemiology* 2005;**58**:1081–8. doi:10.1016/j.jclinepi.2005.09.002
16
Quinn SC, Jamison AM, An J, *et al.* Measuring vaccine hesitancy, confidence, trust and flu vaccine uptake: Results of a national survey of White and African American adults. *Vaccine* 2019;**37**:1168–73.
17
Wilder-Smith AB, Qureshi K. Resurgence of measles in Europe: A systematic review on parental attitudes and beliefs of measles vaccine. *Journal of Epidemiology and Global Health* 2020;**10**:46–58. doi:10.2991/jegh.k.191117.001
18
Hornsey MJ, Lobera J, Díaz-Catalan C. Vaccine hesitancy is strongly associated with distrust of conventional medicine, and only weakly associated with trust in alternative medicine. *Social Science & Medicine* 2020;**255**. doi:10.1016/j.socscimed.2020.113019
19
Stoop N, Hirvonen K, Maystadt J-F. Institutional mistrust and child vaccination coverage in Africa. *BMJ Global Health* 2021;**6**. doi:10.1136/bmjgh-2020-004595
20
Momplaisir F, Haynes N, Nkwihoreze H, *et al.* Understanding drivers of coronavirus disease 2019 vaccine hesitancy among blacks. *Clinical Infectious Diseases* 2021;**in press**. doi:10.1093/cid/ciab102
21
Razai MS, Osama R, McKechnie DGJ, *et al.* Covid-19 vaccine hesitancy among ethnic minority groups. *British Medical Journal* 2021;**372**. doi:10.1136/bmj.n513
22
Pinder RJ, Ferguson J, Møller H. Minority ethnicity patient satisfaction and experience: Results of the National Cancer Patient Experience Survey in England. *BMJ Open* 2016;**6**. doi:10.1136/bmjopen-2016-011938
23
Chauhan A, Walton M, Manias E, *et al.* The safety of health care for ethnic minority patients: A systematic review. *International Journal for Equity in Health*;**19**. doi:10.1186/s12939-020-01223-2
24
Allington D. Do institutional trust and experiences of healthcare explain demographic disparities in coronavirus vaccination uptake? 2021.https://osf.io/dfr3m
25
For peer review only

Wellcome. *Wellcome global monitor: How does the world feel about science and health?* London: : Wellcome Trust 2019.

26

Scottish Government. Inpatient experience survey. 2018.https://www.gov.scot/collections/inpatient-experience-survey/

27

Government U. Population of England and Wales. 2018.https://www.ethnicity-facts-figures.service.gov.uk/uk-population-by-ethnicity/national-and-regional-populations/population-of-england-and-wales/latest

28

R Core Team. *R: A language and environment for statistical computing*. Vienna, Austria: : R Foundation for Statistical Computing 2021. https://www.R-project.org/

29

Revelle W. *Psych: Procedures for psychological, psychometric, and personality research*. Evanston, Illinois: : Northwestern University 2021. https://CRAN.R-project.org/package=psych

30

Charles Dupont FEHJ with contributions from, others. many. *Hmisc: Harrell miscellaneous*. 2021. https://CRAN.R-project.org/package=Hmisc

31

Bischl B, Lang M, Bossek J, *et al. BBmisc: Miscellaneous helper functions for B. Bischl*. 2017. https://CRAN.R-project.org/package=BBmisc

32

Tingley D, Yamamoto T, Hirose K, *et al.* mediation: R package for Causal Mediation Analysis. *Journal of Statistical Software*;**59**:1–38. doi:10.18637/jss.v059.i05

33

Zhang Z, Mai Y. WebPower: Basic and advanced statistical power analysis. 2021. https://CRAN.R-project.org/package=WebPower

---

1. Linear models with standardised predictor and outcome variables.↩︎

For peer review only

STROBE Statement—Checklist of items that should be included in reports of ***cross-sectional studies***

	Item No	Recommendation	Page No
**Title and abstract**	1	(*a*) Indicate the study's design with a commonly used term in the title or the abstract	2
		(*b*) Provide in the abstract an informative and balanced summary of what was done and what was found	2-3
**Introduction**			
Background/rationale	2	Explain the scientific background and rationale for the investigation being reported	3-4
Objectives	3	State specific objectives, including any prespecified hypotheses	5
**Methods**			
Study design	4	Present key elements of study design early in the paper	5-6
Setting	5	Describe the setting, locations, and relevant dates, including periods of recruitment, exposure, follow-up, and data collection	5-6
Participants	6	(*a*) Give the eligibility criteria, and the sources and methods of selection of participants	6
Variables	7	Clearly define all outcomes, exposures, predictors, potential confounders, and effect modifiers. Give diagnostic criteria, if applicable	5-6
Data sources/ measurement	8*	For each variable of interest, give sources of data and details of methods of assessment (measurement). Describe comparability of assessment methods if there is more than one group	5-6
Bias	9	Describe any efforts to address potential sources of bias	7
Study size	10	Explain how the study size was arrived at	5
Quantitative variables	11	Explain how quantitative variables were handled in the analyses. If applicable, describe which groupings were chosen and why	5
Statistical methods	12	(*a*) Describe all statistical methods, including those used to control for confounding	6
		(*b*) Describe any methods used to examine subgroups and interactions	N/A
		(*c*) Explain how missing data were addressed	5
		(*d*) If applicable, describe analytical methods taking account of sampling strategy	N/A
		(*e*) Describe any sensitivity analyses	6

For peer review only - http://bmjopen.bmj.com/site/about/guidelines.xhtml

**Results**			
Participants	13*	(a) Report numbers of individuals at each stage of study—eg numbers potentially eligible, examined for eligibility, confirmed eligible, included in the study, completing follow-up, and analysed	6
		(b) Give reasons for non-participation at each stage	N/A
		(c) Consider use of a flow diagram	N/A
Descriptive data	14*	(a) Give characteristics of study participants (eg demographic, clinical, social) and information on exposures and potential confounders	7-8
		(b) Indicate number of participants with missing data for each variable of interest	6
Outcome data	15*	Report numbers of outcome events or summary measures	6
Main results	16	(*a*) Give unadjusted estimates and, if applicable, confounder-adjusted estimates and their precision (eg, 95% confidence interval). Make clear which confounders were adjusted for and why they were included	7-12
		(*b*) Report category boundaries when continuous variables were categorized	5, 7
		(*c*) If relevant, consider translating estimates of relative risk into absolute risk for a meaningful time period	N/A
Other analyses	17	Report other analyses done—eg analyses of subgroups and interactions, and sensitivity analyses	12-13
**Discussion**			
Key results	18	Summarise key results with reference to study objectives	11-13
Limitations	19	Discuss limitations of the study, taking into account sources of potential bias or imprecision. Discuss both direction and magnitude of any potential bias	14
Interpretation	20	Give a cautious overall interpretation of results considering objectives, limitations, multiplicity of analyses, results from similar studies, and other relevant evidence	13
Generalisability	21	Discuss the generalisability (external validity) of the study results	13-14
**Other information**			
Funding	22	Give the source of funding and the role of the funders for the present study and, if applicable, for the original study on which the present article is based	14

*Give information separately for exposed and unexposed groups.

For peer review only

Note: An Explanation and Elaboration article discusses each checklist item and gives methodological background and published examples of transparent reporting. The STROBE checklist is best used in conjunction with this article (freely available on the Web sites of PLoS Medicine at http://www.plosmedicine.org/, Annals of Internal Medicine at http://www.annals.org/, and Epidemiology at http://www.epidem.com/). Information on the STROBE Initiative is available at www.strobe-statement.org.

For peer review only

For peer review only - http://bmjopen.bmj.com/site/about/guidelines.xhtml

BMJ Open

Trust and experiences of National Health Service healthcare do not fully explain demographic disparities in coronavirus vaccination uptake in the UK: a cross-sectional study

Journal:	*BMJ Open*
Manuscript ID	bmjopen-2021-053827.R1
Article Type:	Original research
Date Submitted by the Author:	06-Jan-2022

Complete List of Authors:	Allington, Daniel; King's College London, Dept. of Digital Humanities<br>McAndrew, Siobhan; University of Bristol, Sociology, Politics, and International Studies<br>Duffy, Bobby; King's College London, Policy Institute<br>Moxham-Hall, Vivienne; King's College London, Policy Institute

**Primary Subject Heading**:	Public health
Secondary Subject Heading:	Infectious diseases
Keywords:	Public health < INFECTIOUS DISEASES, COVID-19, PUBLIC HEALTH

SCHOLARONE™ Manuscripts

I, the Submitting Author has the right to grant and does grant on behalf of all authors of the Work (as defined in the below author licence), an exclusive licence and/or a non-exclusive licence for contributions from authors who are: i) UK Crown employees; ii) where BMJ has agreed a CC-BY licence shall apply, and/or iii) in accordance with the terms applicable for US Federal Government officers or employees acting as part of their official duties; on a worldwide, perpetual, irrevocable, royalty-free basis to BMJ Publishing Group Ltd ("BMJ") its licensees and where the relevant Journal is co-owned by BMJ to the co-owners of the Journal, to publish the Work in this journal and any other BMJ products and to exploit all rights, as set out in our licence.

The Submitting Author accepts and understands that any supply made under these terms is made by BMJ to the Submitting Author unless you are acting as an employee on behalf of your employer or a postgraduate student of an affiliated institution which is paying any applicable article publishing charge ("APC") for Open Access articles. Where the Submitting Author wishes to make the Work available on an Open Access basis (and intends to pay the relevant APC), the terms of reuse of such Open Access shall be governed by a Creative Commons licence – details of these licences and which Creative Commons licence will apply to this Work are set out in our licence referred to above.

Other than as permitted in any relevant BMJ Author's Self Archiving Policies, I confirm this Work has not been accepted for publication elsewhere, is not being considered for publication elsewhere and does not duplicate material already published. I confirm all authors consent to publication of this Work and authorise the granting of this licence.

For peer review only

Trust and experiences of National Health Service healthcare do not fully explain demographic disparities in coronavirus vaccination uptake in the UK: a cross-sectional study

Manuscript submitted: 26/5/2021

Editorial decision received: 13/10/2021

Revised version submitted: 05/01/2022

Daniel Allington (Corresponding Author)

Department of Digital Humanities, King's College London, UK

Address: Department of Digital Humanities, King's College London, Strand, London WC2R 2LS

Email address: daniel.allington@kcl.ac.uk

Siobhan McAndrew

Sheffield Methods Institute, University of Sheffield, Sheffield, UK

Bobby Duffy

Policy Institute, King's College London, London, UK

Vivienne Moxham-Hall

Policy Institute, King's College London, London, UK

Original word count: 4269

Revised word count: 5234

For peer review only

Trust and experiences of National Health Service healthcare do not fully explain demographic disparities in coronavirus vaccination uptake in the UK: a cross-sectional study

Daniel Allington, Siobhan McAndrew, Bobby Duffy, and Vivienne Moxham-Hall

Initial draft submitted: 26/5/2021

Revised draft submitted: 5/1/2022

For peer review only

Abstract

Objectives

To test whether demographic variation in vaccine hesitancy can be explained by trust and healthcare experiences.

Design

Cross-sectional study.

Setting

Data collected online in April 2021.

Participants

Data collected from 4885 UK-resident adults, of whom 3223 had received the invitation to be vaccinated against the novel coronavirus and could therefore be included in the study. 1629 included participants identified as female and 1594 as male. 234 identified as belonging to other than white ethnic groups while 2967 identified as belonging to white ethnic groups.

Primary and secondary outcome measures

Uptake of coronavirus vaccination.

Results

Membership of an other than white ethnic group (AOR = 0.53, 95% CI [0.35, 0.84], p = .005) and age (AOR = 1.61, 95% CI [1.39, 1.87], p < .001 for a 1 SD change from the mean) were the only statistically significant demographic predictors of vaccine uptake. After controls for NHS (National Health Service) healthcare experiences and trust in government, scientists, and medical professionals, the effect associated with membership of an other than white ethnic group appears more marginal (AOR = 0.61, 95% CI [0.38, 1.01], p = .046), while the effect associated with age remains virtually unchanged. Exploratory analysis suggests that NHS healthcare experiences mediate 24% (95% CI [8%, 100%], p = .024) of the association between ethnicity and uptake, while trust mediates 94% (95% CI [56%, 100%], p .001) of the association between NHS healthcare experiences and uptake.

Conclusions

Members of other than white ethnic groups report inferior NHS healthcare experiences, potentially explaining their lower reported trust in government, scientists, and medical professionals. However, this does not fully explain the ethnic gap in coronavirus vaccination uptake.

Trial registration

Data collection and confirmatory analysis were pre-registered with the Center for Open Science (osf.io/56txk).

Article summary

Strengths and limitations of this study

- The sample was large, providing high statistical power, and was designed for representativeness of the UK adult population
- Standard measures of trust and healthcare experiences were used, providing confidence that these variables have been measured robustly
- However, it was not possible to obtain a probability sample, and there was limited representation within the sample both of people who did not take up the invitation to be vaccinated and of members of other than white ethnic groups (given that both of these are relatively small minorities in the overall UK population)
- Moreover, given the younger age profile of other than white ethnic groups in the UK, it is difficult to disentangle the effects associated with age from the effects associated with ethnicity

For peer review only

1 Introduction

The problem of vaccine hesitancy, defined as 'delay in acceptance or refusal of vaccination despite availability of vaccination services' [1], has been recognised as a major global health problem for several years [2,3]. However, it gained particular importance during the COVID-19 public health emergency: early in the pandemic, it was estimated that as much as 82% of a population might need vaccination or natural immunity against the disease before herd immunity could be reached [4], but one survey indicated that less than 72% of the British population might be willing to accept vaccination against SARS-CoV-2, the novel coronavirus which causes COVID-19 [5]. Multiple surveys found demographic disparities in British respondents' expressed likelihood of vaccine uptake, with younger, less educated respondents, women, members of lower income households, and members of other than white ethnic groups all tending to express a lower probability of accepting the offer of vaccination [6–8]. The finding with regard to ethnicity has been of particular concern, given higher fatality rates for COVID-19 among members of minority ethnic groups [9]. Actual uptake has been far higher than these surveys would have predicted, with one longitudinal study finding that 99% of those who had stated in December 2020 that they would accept vaccination, and 86% of those who had stated at the same point that they would not or were unsure, stated in February 2021 either that they would accept vaccination or that they had already done so [10]. However, despite this encouraging trend, demographic disparities appeared to persist [11], albeit at a reduced level, with youth and membership of other than white ethnic groups now being the strongest predictors of coronavirus vaccine hesitancyuptake in the UK [10], such that by 13 January 2021, 42.5% of white people aged over 80 but not resident in care homes, but only 20.5% of black people in the same category, had been vaccinated [12]. The potential health consequences of incomplete vaccination coverage were recently highlighted by the official claim that the majority of people hospitalised as a result of a COVID-19 outbreak in the English town of Bolton were unvaccinated yet eligible to be vaccinated [13].

The possibility that lower trust in government, scientists, and the medical system might lead to reduced coronavirus vaccine uptake among members of minority ethnic communities had already been noted before any survey data had been collected that might serve to test such a hypothesis [14]. Indeed, many studies have found a relationship between vaccine hesitancy and mistrust in medicine and other relevant institutions, both on an individual level [15–18] and on a national level [19], with regard to vaccination against other diseases. Evidence now exists to suggest that coronavirus vaccine hesitancy among African Americans may be driven by mistrust in the medical establishment (itself resulting from awareness of past mistreatment and unethical practices on the part of medical professionals and researchers), as well as by perceptions of racism in the political system [20]. A systematic review of factors potentially explaining coronavirus vaccination uptake within minority ethnic communities in the UK found one study which identified '[l]ogistical and practical barriers, such as the location of vaccine centres and having to use public transport' as a potential driver of lower vaccine uptake rates, six which attributed reduced uptake to 'mistrust including pre-existing lower scientific or medical trust, conspiracy suspicions and attitudes', and four which found that 'people from minority ethnic backgrounds were more likely than White British groups to have received misinformation encouraging them not to have the vaccine' [21].

Some experts have attributed heightened levels both of coronavirus misinformation and of coronavirus vaccine hesitancy among members of some minority ethnic communities in the UK to mistrust which they argue is in turn driven by experiences of racism, discrimination, and exclusion [22]. As members of minority

For peer review only

ethnic groups report lower patient satisfaction and worse healthcare experiences in the UK [23], and, on an international level, appear to suffer from 'higher incidences of healthcare associated infections, dosing errors, [adverse drug events], and complications resulting from their care' [24], it would seem plausible that systematically poorer healthcare experiences may act as a driver of ethnic disparities in coronavirus vaccine uptake, alongside strategic and situational trust. Moreover, it would appear plausible that some factors potentially accounting for lower vaccine uptake within ethnic minority populations (including lower trust resulting from poor healthcare experiences and awareness of past malpractice) might also be associated with lower rates of vaccine uptake among other groups, potentially including women, young people, less highly educated people, and members of low income households. On the other hand, one UK study has found that '[s]ocio-demographics do not explain vaccine hesitancy to any helpful degree' and that the major predictors of coronavirus vaccine hesitancy are 'excessive mistrust' and a lack of 'positive healthcare experiences' [5]. In context of the urgent need to understand and resolve disparities in vaccine coverage, using both quantitative and qualitative data as appropriate, these findings require interrogation through replication. For example, it might be that demographic variables only fail to explain variation following controls for trust and healthcare experiences because these arguably more proximal variables act to mediate the effects associated with certain demographic variables. Moreover, the use of more standard measures for trust and healthcare experiences would give greater confidence, as would pre-registration of hypotheses.

This article reports on a pre-registered study designed in order to test for demographic predictors of coronavirus vaccination uptake in the UK, both before and after controls for healthcare experiences and for trust in the principal institutions associated with the UK's coronavirus vaccination programme, i.e. government, scientists, and medical professionals [25]. As the UK's vaccination programme is delivered by National Health Service (NHS) bodies in England, Scotland, Wales, and Northern Ireland, healthcare experience items focused specifically on experiences of NHS healthcare. In order to provide the most robust test possible, established instruments have been used to measure both trust and healthcare experiences, rather than the novel measures employed in the study which found demographic variables not to be predictive [5].

2 Hypotheses

Given the above observations regarding published findings of existing research, the following expectations were formed:

H1. Coronavirus vaccination uptake will be lower amongst female respondents
H2. Coronavirus vaccination uptake will be lower amongst younger respondents
H3. Coronavirus vaccination uptake will be lower amongst respondents of other than white ethnic groups
H4. Coronavirus vaccination uptake will be lower amongst less educated respondents
H5. Coronavirus vaccination uptake will be lower amongst respondents from lower-income households
H6. Coronavirus vaccination uptake will be positively associated with trust in government, medical professionals, and scientists
H7. Coronavirus vaccination uptake will be positively associated with positive experiences of healthcare

3 Methods

For peer review only - http://bmjopen.bmj.com/site/about/guidelines.xhtml

3.1 Study design

Trust was measured using Wellcome Global Health Monitor items Q11B, Q11E, Q14A, and Q15a [26]. Experiences of healthcare were measured using items adapted from the Scottish Government Inpatient Experience Survey [27]. Responses to individual questions were numerically recoded and averaged to provide a single measure of trust and a single measure of experience of NHS healthcare. To facilitate analysis of individual trust and healthcare items, responses to these items were separately dichotomised for use in additional partial models, with the top two levels recoded as true and others recoded as false (this pre-registered arbitrary cut-off point being chosen because it could be applied to all necessary variables regardless of how many levels they had).

Education was operationalised as highest qualification received, which was dichotomised in order to distinguish degree-level (and equivalent) qualifications (including undergraduate and postgraduate degrees) from lower levels of qualification, including lack of all formal qualifications. Ethnic group was measured using the categories employed in the 2011 UK census, which were subsequently dichotomised through coding as white or other than white, and household income was measured on an ordinal scale and dichotomised through coding as below median and median or above (for the sample). Dichotomisation of ethnicity has the unfortunate effect of homogenising the experiences of diverse minority groups, but was necessary because of the very small size of all minority groups when considered individually. Gender was measured by asking respondents whether they identified as 'male', 'female', or 'in another way'. As it was anticipated that relatively few respondents would not identify with one or other of the two canonical genders, gender was operationalised as a binary variable by reclassifying both 'male' and 'other' respondents as 'other than female'. This step proved to be unnecessary, as no participants in the sample identified either as male or as female. However, because it had been pre-registered, the transformation was retained.

The dependent variable is a dichotomous variable representing the condition of having been vaccinated against the novel coronavirus (in the event that the respondent has been invited to be vaccinated) or of being about to attend an appointment to be vaccinated (in the event that a respondent was surveyed immediately after being invited to be vaccinated, and for that reason has not been vaccinated yet).

All variables were measured through self-report, as the questionnaire was completed online.

In calculating indices, missing values were ignored. Cases with missing data in relation to specific variables were excluded from all analyses featuring those variables.

3.2 Data collection

Although there is no longitudinal aspect to the study reported here, the data collection formed part of a longitudinal study in which each wave involves collection both from recontactees and from new participants. Respondents were sampled at random from an Ipsos MORI recruited panel, with pre-stratification in order to produce representativeness of the UK adult population on age, gender, geographical region, and working status. Where representativeness was not achieved, additional respondents were obtained by Ipsos MORI on a quota basis from panels maintained by other data providers. Following standard practice in the British polling industry, no quotas were applied with regard to ethnicity. However, overall proportions of white and other than white respondents within the sample were nonetheless broadly representative of the UK population (see below). Data were collected from respondents who have not been offered vaccination against

For peer review only - http://bmjopen.bmj.com/site/about/guidelines.xhtml

the novel coronavirus, but data on those respondents were excluded from this particular study, as they could not be used to test hypotheses about vaccine uptake.

Data collection was carried out online by Ipsos MORI. Fieldwork was launched on 01 April 2021 and completed on 16 April 2021. Informed consent was obtained from all participants. Demographic weights were calculated post-collection by Ipsos MORI using the random iterative method on the basis of education and geographical region and of gender interlocked with age, NRS social grade, and working status. Weights were calculated before exclusion of cases where respondents had not yet been invited to receive coronavirus vaccination, as population margins for invited people specifically are not available. Data were collected from a total of 4885 respondents, of whom 1662 were excluded due to not having received the invitation to be vaccinated. Table 1 provides a breakdown of the full sample by ethnicity, using the full range of UK census categories (note that it was possible to refuse consent for collection of data on ethnicity whilst giving consent to participate in the study as a whole). In comparison with the 2011 census, there was lower representation of the majority ethnic group, i.e. White English / Welsh / Scottish / Northern Irish / British, than in the 2011 census, along with greater representation of individuals of Other White background, although these discrepancies might plausibly be attributed to actual population changes in the intervening decade (findings of the 2021 census have not yet been released); other groups are so small that differences in representation are probably not meaningful to discuss (e.g. 2% Indian in this sample versus 2.5% in the 2011 census). Numbers of included cases with missing values for each variable are listed in Table 2, with a breakdown by dichotomised ethnicity. This shows that there was a higher rate of missing data for NHS healthcare among white respondents than among other than white respondents, amounting to roughly 3% of included cases in the former case (in large part because the individuals concerned did not use NHS healthcare services), and a higher rate of missing data for vaccine uptake among other than white respondents than among white respondents, amounting to roughly 1% of included cases in the former case.

Table 1: Ethnic groups in full sample

**Ethnic group**	**n**	
White English / Welsh / Scottish / Northern Irish / British	4009	82%
White Irish	49	1%
White Gypsy or Irish Traveller	1	0%
Any other White background	319	7%
White and Black Caribbean	22	0%
White and Black African	12	0%
White and Asian	32	1%
Any other Mixed / multiple ethnic background	22	0%
Indian	78	2%
Pakistani	77	2%
Bangladeshi	39	1%
Chinese	40	1%
Any other Asian background	22	0%
African	44	1%
Caribbean	33	1%
Any other Black / African / Caribbean background	9	0%
Arab	9	0%
Any other ethnic group, please write in	14	0%
Prefer not to answer	42	1%
Consent not granted	12	0%

Table 2: Included cases with missing values

	**Age**	**Gender**	**Degree**	**Other than white**	**Low income**	**Trust**	**NHS experiences**	**Uptake**
Overall	0	0	0	22	305	8	124	46
White	0	0	0		267	5	121	27
Other than white	0	0	0		26	1	2	14

3.3 Statistical analysis

The sample was treated as equivalent to a random sample for analytic purposes. Hypotheses 1-7 were tested through creation of binomial logit models with vaccine acceptance as the dependent variable. Of most relevant interest are a partial model using only demographic predictors, and the full model featuring demographic predictors and aggregate measures of trust and of experiences of NHS healthcare. Both of these models were pre-registered; as explained in the pre-registration document, the full model was considered the definitive test of the hypotheses, with other models provided for information. To further probe the findings of the confirmatory analysis (see below), two further partial models were created without having been pre-registered. These included one featuring demographic predictors and aggregate measures of trust only and one featuring demographic predictors and aggregate measures of NHS healthcare only. In order to study the predictiveness of individual items, two further additional pre-registered models were created, one featuring demographic predictors and dichotomised responses to trust items as predictors, and the other featuring demographic variables and dichotomised responses to healthcare experience items as predictors, although (as noted above) it was the full model that was considered the definitive test. To further understand the unexpected findings of the confirmatory analyses, mediation analyses were conducted on an exploratory basis using non-parametric bootstrapping with 10000 repetitions in order to test for theoretically plausible mediatory relationships suggested by the finding that certain variables were significantly predictive in partial models but not in the full model. Being developed in response to the findings of the pre-registered analyses, these mediation analyses are detailed below. (The unusually high number of repetitions was chosen in order to reduce random variation, despite the increase in demands on computer processing power). Coefficients for all confirmatory and exploratory analyses are reported both as estimates and as 95% confidence intervals.

3.4 Power analysis

Power analysis confirms that the sample was sufficiently large to detect even very small effects, at least in the pre-registered models. Given a sample size of 2773 (i.e. the number of observations which were sufficiently complete to be fitted in the full model) and a cut-off of $p < .050$ (two-tailed), an association so weak as to reduce the probability of vaccine uptake only from 95% to 91% across the entire range of a predictor variable could be detected with approximately 98% power.

3.5 Patient and public involvement

No patient involved.

4 Results

For peer review only - http://bmjopen.bmj.com/site/about/guidelines.xhtml

4.1 Descriptive statistics and internal reliability

Table 3: Descriptive statistics: demographic variables

		Age		Gender		Degree		Ethnic group[a]		Household income[b]		
	**n**	**M**	**SD**	**F.**	**M.**	**Yes**	**No**	**W.**	**O.**	**L.**	**M.**	**H.**
**All invited**												
Raw	3223	52.00	14.64	51%	49%	39%	61%	93%	7%	43%	19%	38%
Weighted		51.89	14.70	51%	49%	28%	72%	92%	8%	44%	20%	37%
**Uptake: yes**												
Raw	2990	52.78	14.40	51%	49%	40%	60%	94%	6%	43%	19%	38%
Weighted		52.70	14.48	51%	49%	28%	72%	93%	7%	44%	19%	37%
**Uptake: no**												
Raw	187	44.10	13.83	50%	50%	34%	66%	85%	15%	42%	23%	35%
Weighted		44.01	13.65	49%	51%	24%	76%	84%	16%	42%	24%	33%

[a] W (white) ethnic groups comprise white British, English, Welsh, Scottish, Northern Irish, Irish, Gypsy or Irish Traveller, and any other white background; O (other than white) ethnic groups comprise all other ethnic categories used in the 2011 UK census, including mixed ethnicities

[b] L (low) = £24,999 or less; M (medianfor included participants) = £25,000 - £34,999; H (high) = £35,000 or more

Cronbach's alpha and Guttman's lambda 6 were used to assess internal reliability of indices. The reliability of items used to measure experiences of NHS healthcare was assessed as $\alpha = .88$, $\lambda_6 = .87$, which indicates a high level of internal reliability. The reliability of items used to measure trust was assessed as $\alpha = .69$, $\lambda_6 = .66$, which would be a suboptimal level of internal reliability for a conventional Likert scale but likely reflects real differences in levels of trust for different institutions (especially given that government trust may be acting at least partly as a proxy for political preference).

For peer review only - http://bmjopen.bmj.com/site/about/guidelines.xhtml

Table 4: Age profile of dichotomised ethnic groups among included respondents

**Ethnicity**	**n**	**M**	**SD**
White	2967	53.14	14.12
Other than white	234	38.15	13.91

Table 5: Descriptive statistics: indices

		**NHS exp.**		**Trust**	
	**n**	**M**	**SD**	**M**	**SD**
**All invited**					
Raw	3223	4.06	0.71	3.09	0.52
Weighted		4.05	0.71	3.08	0.53
**Uptake: yes**					
Raw	2990	4.08	0.69	3.12	0.48
Weighted		4.08	0.69	3.12	0.48
**Uptake: no**					
Raw	187	3.70	0.92	2.64	0.77
Weighted		3.69	0.94	2.64	0.78

Table 6: Key variables: bivariate correlations

		**1**	**2**	**3**	**4**	**5**	**6**	**7**	**8**
1	Age		-.01	-.27	.11	-.09	.10	.06	.14
2	Female	-.01		-.04	.05	.05	.01	.05	.00
3	Other than white	-.27	-.04		-.01	.03	-.13	-.08	-.08
4	Low income	.11	.05	-.01		-.21	-.03	-.06	.00
5	Degree	-.09	.05	.03	-.21		.04	.04	.03
6	NHS experiences	.10	.01	-.13	-.03	.04		.47	.13
7	Trust	.06	.05	-.08	-.06	.04	.47		.22
8	Uptake	.14	.00	-.08	.00	.03	.13	.22	

Table 3 shows weighted and unweighted demographic descriptive statistics, first for all included respondents, and then for respondents who did and did not accept the invitation to be vaccinated against the novel coronavirus. Although respondents from households with income at or above the median for the sample were treated as a single group for modelling purposes, they are disaggregated here. It will be noted that the percentage of white respondents is higher than in the raw sample. However, this was to be expected, as inclusion was limited to individuals who had been offered vaccination in a context where vaccination had been offered in descending order of age, and where other than white ethnic groups have a younger age profile [28]. Table 4 shows that this is also the case for included respondents in the current study. It is acknowledged that the number of other than white respondents was small in absolute terms, but it is noted that collection of a larger number would have required deliberate oversampling (and therefore a less representative sample).

The mean age for respondents who had been invited to be vaccinated yet had not taken up the invitation is just under nine years younger than the mean age for respondents who had been vaccinated. Percentages of male and female and respondents in each group are effectively identical. Percentages with low (below median) household incomes for the sample are effectively identical, but the weighted percentage of

respondents with high (above median) household incomes for the sample is 4 points lower among those who did not accept the invitation to be vaccinated (i.e. the proportion was about 0.9 times as high). This suggests that the effect of income may be masked in the confirmatory analysis presented in this article, as dichotomisation by separating low income households from median and high income households was a pre-registered data transformation: a point to which we shall return below. The weighted percentage of respondents educated to degree level was 4 points lower among those who did not accept the invitation to be vaccinated (i.e. it was about 0.9 times as high). The weighted percentage of respondents belonging to other than white ethnic groups was 9 points higher among those who did not accept the invitation to be vaccinated (i.e. it was about 2.3 times as high). This is not a large difference: by the time of data collection, 94% and 87% of invited members of white and other than white ethnic groups within the sample who had received the invitation to be vaccinated had taken it up, which implies that the great majority of members of all ethnic groups had done so. However, it is consistent with the observation, discussed above, that uptake rates have been lower amongst members of other than white ethnic groups.

Table 5 shows weighted and unweighted indices of trust and NHS healthcare experiences, first for the whole sample, and then for respondents who did and did not accept the invitation to be vaccinated against the novel coronavirus. Mean scores for members of the latter group were lower on both indices. Table 6 shows unweighted product-moment correlations between all pairs of variables. There was a strong correlation between indices of trust and NHS healthcare experiences (r = .47), but NHS healthcare experiences were less strongly correlated with vaccine uptake (r = .13) than trust was (r = .22), while trust was less strongly correlated with membership of an other than white ethnic group (r = -.08) than NHS healthcare experiences were (r = -.13). Age was negatively correlated with membership of an other than white ethnic group (r = -.27) and with degree level education (r = -.09), but positively correlated with membership of a low income household (r = .11) and with vaccination uptake (r = .14).

For peer review only

Table 7: Partial models and full model

	AOR	2.5%	97.5%	p
**Demographics only**				
Age	1.61	1.39	1.87	<.001
Female	0.96	0.71	1.30	.808
Other than white	0.53	0.35	0.84	.005
Low income	1.02	0.75	1.40	.883
Degree	1.30	0.92	1.87	.140
**NHS experiences**				
Age	1.56	1.34	1.83	<.001
Female	0.98	0.71	1.34	.882
Other than white	0.65	0.41	1.04	.064
Low income	1.09	0.79	1.51	.591
Degree	1.29	0.91	1.87	.167
NHS experiences	1.46	1.27	1.67	<.001
**Trust**				
Age	1.57	1.34	1.85	<.001
Female	0.89	0.65	1.23	.490
Other than white	0.61	0.39	0.98	.037
Low income	1.12	0.81	1.56	.491
Degree	1.18	0.82	1.71	.380
Trust	2.06	1.80	2.36	<.001
**Full model**				
Age	1.57	1.33	1.86	<.001
Female	0.92	0.66	1.28	.615
Other than white	0.61	0.38	1.01	.046
Low income	1.16	0.83	1.63	.379
Degree	1.22	0.84	1.80	.297
NHS experiences	1.02	0.86	1.20	.820
Trust	2.02	1.72	2.37	<.001

For peer review only

Table 8: Individual trust and NHS healthcare experience items (dichotomised)

	AOR	2.5%	97.5%	p
**NHS experiences**				
Age	1.51	1.30	1.77	<.001
Female	0.91	0.67	1.24	.556
Other than white	0.64	0.41	1.01	.049
Low income	1.03	0.75	1.41	.848
Degree	1.24	0.88	1.79	.226
Respect	0.99	0.61	1.59	.967
Care	1.17	0.72	1.89	.513
Needs	1.45	0.96	2.18	.077
Trust staff	1.62	1.01	2.58	.045
Understand	1.37	0.89	2.11	.148
Spiritual	0.76	0.54	1.07	.113
**Trust**				
Age	1.52	1.30	1.78	<.001
Female	0.91	0.67	1.24	.552
Other than white	0.68	0.43	1.09	.097
Low income	1.12	0.82	1.54	.487
Degree	1.20	0.84	1.72	.322
Trust UK gov.	1.48	1.07	2.07	.021
Trust sci. uni.	1.77	1.12	2.77	.013
Trust sci. corp.	1.94	1.34	2.78	<.001
Trust medics	2.81	1.74	4.49	<.001

4.2 Confirmatory analysis

Table 7 presents adjusted odds ratios (with 95% confidence intervals) for dichotomous variables (female gender, membership of an other than white ethnic group, membership of a low income household, and education to undergraduate or postgraduate degree level) and for increases of 1 SD in numeric variables (i.e. age and the indices for trust and NHS healthcare experiences). Age (AOR = 1.61, 95% CI [1.39, 1.87], $p < .001$) and membership of an other than white ethnic group (AOR = 0.53, 95% CI [0.35, 0.84], p = .005) were the only statistically significant predictors in the purely demographic model. Thus, the null hypothesis can be rejected with regard to H2 and H3, but not with regard to H1, H4, and H5.

Controlling for indices of trust and of NHS healthcare experiences diminishes the effect associated with membership of an other than white ethnic group from AOR = 0.53, 95% CI [0.35, 0.84], p = .005 to AOR = 0.61, 95% CI [0.38, 1.01], p = .046. This suggests that some of the effect associated with ethnicity may be mediated by one or both of those additional variables. However, the same cannot be said for age. Indeed, the null hypothesis can only be rejected for H3 with regard to this model at $p < .050$, but the coefficient and p-value relevant to H2 are virtually unchanged relative to the demographic-only model. Mroreover, while there was a significant effect associated with trust in the full model (AOR = 2.02, 95% CI [1.72, 2.37], $p < .001$), there was no effect associated with NHS healthcare experiences (AOR = 1.02, 95% CI [0.86, 1.2], p = .820).

For peer review only - http://bmjopen.bmj.com/site/about/guidelines.xhtml

Thus, the null hypothesis can be rejected with regard to H6, but not with regard to H7.

This finding would appear to be in contradiction of the claim that vaccine hesitancy is largely explained by trust alongside healthcare experiences, and that demographic predictors have little importance. Partial models excluding trust or healthcare experiences are also presented in Table 7. As the effect associated with NHS healthcare experiences in the partial model excluding trust is very highly significant (AOR = 1.46, 95% CI [1.27, 1.67], p < .001), albeit lower than the effect associated with trust in the partial model excluding NHS healthcare experiences (AOR = 2.06, 95% CI [1.8, 2.36], p < .001, AOR = 2.06, 95% CI [1.8, 2.36], p < .001), it seems plausible that the effect of NHS healthcare experiences on uptake may be mediated by trust: a possibility to which we shall return below.

Table 8 presents adjusted odds ratios (with 95% confidence intervals) for increases of 1 SD in age and for dichotomous variables, including dichotomised trust and NHS healthcare items as individual predictors. Examination of coefficients associated with those items facilitates assessment of their individual predictive contribution. All effects associated with trust items are statistically significant, especially those associated with trust in medical professionals (AOR = 2.81, 95% CI [1.74, 4.49], p < .001) and with scientists working in the private sector (AOR = 1.94, 95% CI [1.34, 2.78], p < .001). Thus, high trust in any of the institutions mentioned was associated with increased likelihood of uptake, even after controlling for level of trust in all the others, plus demographic variables. Interestingly, the only significant effect associated with an individual NHS healthcare experience item after controlling for all the other items and for demographic variables was that associated with having felt able to trust the staff (AOR = 1.62, 95% CI [1.01, 2.58], p = .045). In relation to these models, the null hypothesis is still rejected with regard to H6, and may be partially rejected with regard to H7.

4.3 Exploratory analysis

The above findings were suggestive of theoretically plausible mediated associations, i.e. that experiences of NHS healthcare might be indirectly associated with uptake via trust, that ethnicity might be indirectly associated with trust via experiences of NHS healthcare, and that ethnicity might therefore be indirectly associated with uptake via both of the aforementioned. For this reason, additional mediation analyses were carried out on an exploratory basis (i.e. without having been pre-registered). All of these analyses involved the same demographic controls used in the confirmatory analyses.

There were three findings. Firstly, the relationship between membership of an other than white ethnic group and trust appears to be 100% (95% CI [48%, 100%], p = .040) mediated by NHS healthcare experiences.[1] Secondly, the relationship between NHS healthcare experiences and uptake appears to be 94% (95% CI [56%, 100%], p < .001) mediated via trust. Thirdly, the association between membership of an other than white ethnic group and uptake appears to be 24% (95% CI [8%, 100%], p = .024) mediated via NHS healthcare experiences (trust was not included in this model, given the second finding). This mediating effect may plausibly be taken to account for the reduction in the effect associated with ethnicity between the demographic-only model and the full confirmatory model.

While these findings are exploratory rather than confirmatory, they add weight to the above interpretation of the confirmatory findings. On the one hand, they suggest that positive experiences of NHS healthcare may be associated with higher vaccine uptake largely for the reason that they have a positive relationship with trust in relevant institutions, and that membership of an other than white ethnic group is associated with lower

For peer review only - http://bmjopen.bmj.com/site/about/guidelines.xhtml

trust in those same institutions for the reason that experiences of NHS healthcare are less positive among members of such groups. On the other hand, these findings also suggest that those intermediary relationships can only account for some of the association between ethnicity and vaccine uptake. Most of that association remains unexplained, as does the association between age and uptake.

5 Discussion

The finding that female gender, below degree-level education, and below median income do not predict uptake contradicts several studies which measured vaccine hesitancy before the UK vaccination campaign had got underway [6–8,10]. This discrepancy may be interpreted in terms of an attitude change potentially attributable to public health communication, word of mouth discussion of coronavirus vaccination itself, or many other sources (although it is also possible that members of certain groups simply under-estimated their own likelihood of accepting vaccination).

The finding that age and ethnicity do, however, predict uptake even after controls for trust and NHS healthcare experiences is in contradiction of the earlier finding that vaccine hesitancy is largely explained by mistrust and poor experiences of healthcare, to such an extent that demographic variables are not predictive once those more proximal variables are controlled for [5]. Non-replication of that finding might be accounted for by the timeframe (data collected after the vaccination programme had begun), outcome measure (actual uptake rather than expected uptake), predictor measures (those used here both for trust and for healthcare experiences were more standard), or analytic approach (pre-registered models rather than exploratory modelling). Age is here found to remain a powerful predictor of uptake even after those controls, and ethnicity is also found to predict uptake, albeit that this effect appears weaker after the same controls. The finding that age is positively associated with uptake replicates an earlier large cohort study [10], and is consistent with the several studies which have found a negative association between age and coronavirus vaccine hesitancy [6-8]. The reduction in strength of association for ethnicity following controls appears to be the result of mediation by NHS healthcare experiences, via trust in the institutions involved in developing and delivering the vaccine – which provides support for the argument that lower vaccine uptake in ethnic minority populations might be driven via lower trust resulting from worse healthcare experiences [22]. However, the mediation effect is small, appearing to account only for about a quarter of the variation in uptake which was found to be associated with ethnicity in the demographic-only model. To summarise, this study finds evidence that uptake of coronavirus vaccination is lower among members of other than white ethnic groups, that NHS healthcare experiences have a relationship with vaccine uptake that is mediated by trust, and that the lower trust in medics, scientists, and government among members of other than white ethnic groups is mediated by their worse experiences of NHS healthcare. However, it does not find evidence that worse healthcare experiences or lower trust can explain more than a small part of the ethnicity gap in coronavirus vaccine uptake.

This finding (or non-finding) suggests that lower rates of vaccination uptake among members of other than white ethnic groups are likely to have drivers other than or in addition to mistrust of medical, scientific, and other authorities and to negative experiences of healthcare. Experiences of healthcare explain little or no variation in uptake beyond that which is explained by trust, their association with uptake being almost entirely mediated by the latter. In some ways, this is encouraging: had the effect associated with ethnicity been found to be entirely explicable in terms of worse experiences of healthcare, then prospects of reducing it in the short term — and especially under pandemic conditions — would appear very bleak. However, the fact

For peer review only - http://bmjopen.bmj.com/site/about/guidelines.xhtml

that so much variance remains unexplained is in itself a cause for concern, and highlights the urgent need for further research to test the mediating role of other variables, alongside qualitative research aiming to uncover the detailed mechanisms by which ethnicity and other demographic variables might be related to uptake. Possible candidate mediators involve access barriers with regard to coronavirus vaccination services [22], as well as sources of information, conspiracy beliefs about the novel coronavirus, and attitudes to vaccination in general [8]. Moreover, the finding that ethnic disparities in trust — a vital component in public health — could be so closely related to poorer reported experiences of NHS healthcare should serve as a reminder of the very real problem of ethnic disparities in healthcare experiences and outcomes, whether those disparities can be assumed to have a population-level impact on coronavirus vaccine uptake or not.

6 Limitations of this study

This study relies on self-report data collected, and as such, it is dependent both on respondents' candour and on their recall. Also, while much effort has been made to produce a representative sample, it was not possible to obtain a probability sample. Moreover, as noted above, the focus on low income as a predictor (to which the researchers were committed by the pre-registration document) may have concealed the importance of high income as a predictor. In addition, the data collected here provide no basis on which to ascertain whether the effect associated with age reflects a genuine tendency towards lower uptake or rather results simply from the UK's vaccination schedule: because older people were invited first, they will have had more time in which to be vaccinated (although as noted above, other studies have found a negative association between age and vaccine hesitancy). Lastly, while the researchers were working with a relatively large sample, it included only 187 individuals who did not take up the invitation to be vaccinated, and only 234 members of other than white ethnic groups. Given that any effects associated with ethnicity must be disentangled from those associated with age, which in this context functions as a confound (both because age had a stronger association with uptake than ethnicity and because members of other than white ethnic groups were on average younger than members of white ethnic groups in the sample, as in the wider UK population), special caution must therefore be exercised in interpreting the results presented here. Further study of relationships such as those focused on in this study, for example looking for variables that might mediate the relationship between age and uptake, or that might explain the currently unexplained majority of the relationship between ethnicity and uptake, might therefore benefit either from an even larger sample or from deliberate over-sampling of individuals who have not taken up the invitation to be vaccinated, and perhaps also of members of specific demographic groups.

For peer review only

7 Technical note

The analysis presented here was carried out using R v 4.0.5 [29], with use of the following packages: psych v. 2.1.3 [30] for calculation of internal reliability of scales, Hmisc v. 4.5.0 [31] for calculation of weighted standard deviations, BBmisc v. 1.11 [32] for standardisation of numeric variables, mediation v. 4.5.0 [33] for mediation analysis, and WebPower v. 0.6 [34] for power analysis.

8 Contributorship statement

Daniel Allington, Siobhan McAndrew, Bobby Duffy, and Vivienne Moxham-Hall were jointly responsible

For peer review only - http://bmjopen.bmj.com/site/about/guidelines.xhtml

for design of the data collection instrument. Daniel Allington planned, pre-registered, and executed the analysis presented here, and wrote this article together with Siobhan McAndrew, who has overall leadership of the project within which this research took place. Revisions of the article following peer review were implemented by Daniel Allington.

9 Funding statement

This research was supported by the Economic and Social Research Council, ES/V 015494/1.

10 Ethics approval statement

King's College London ethics committee, MRA-20/21-21420.

11 Data sharing statement

Data will be made available following the conclusion of the project's period of funding.

12 Competing interests statement

The authors have no competing interests to report.

For peer review only

References

1
MacDonald NE, SAGE Working Group on Vaccine Hesitancy. Vaccine hesitancy: Definition, scope and determinants. *Vaccine* 2015;**33**:4161–4.
2
Dubé E, Gagnon D, Nickels E, *et al.* Mapping vaccine hesitancy—country-specific characteristics of a global phenomenon. *Vaccine* 2014;**32**:6649–54. doi:10.1016/j.vaccine.2014.09.039
3
WHO. Ten threats to global health in 2019. World health organisation. 2019.https://www.who.int/news-room/spotlight/ten-threats-to-global-health-in-2019
4
Sanche S, Lin Y, Xu C, *et al.* High contagiousness and rapid spread of Severe Acute Respiratory Syndrome Coronavirus 2. *Emerging Infectious Diseases* 2020;**26**:1470–7. doi:10.3201/eid2607.200282
5
Freeman D, Loe BS, Chadwick A, *et al.* COVID-19 vaccine hesitancy in the UK: The Oxford coronavirus explanations, attitudes, and narratives survey (OCEANS) II. *Psychological Medicine* 2020;1–5. doi:10.1017/S0033291720005188
6
Callaghan T, Moghtaderi A, Lueck JA, *et al.* Correlates and disparities of COVID-19 vaccine hesitancy. *Social Science & Medicine* 2021;**272**. doi:10.1016/j.socscimed.2020.113638
7
Allington D, McAndrew S, Moxham-Hall VL, *et al.* Media usage predicts intention to be vaccinated against SARS-CoV-2 in the US and the UK. *Vaccine* 2021;**39**:2595–603. doi:10.1016/j.vaccine.2021.02.054
8
Allington D, McAndrew S, Moxham-Hall V, *et al.* Coronavirus conspiracy suspicions, general vaccine attitudes, trust, and coronavirus information source as predictors of vaccine hesitancy among UK residents during the COVID-19 pandemic. *Psychological Medicine* 2021;1–7. doi:10.1017/S0033291721001434
9
ONS. *Updating ethnic contrasts in deaths involving the coronavirus (COVID-19), England and Wales: Deaths occurring 2 March to 28 July 2020*. Office for National Statistics 2020. https://www.ons.gov.uk/peoplepopulationandcommunity/birthsdeathsandmarriages/deaths/articles/updatingethniccontrastsindeathsinvolvingthecoronaviruscovid19englandandwales/deathsoccurring2marchto28july2020
10
Byrne T, Patel P, Shrotri M, *et al.* Trends, patterns and psychological influences on COVID-19 vaccination intention: Findings from a large prospective community cohort study in England and Wales (Virus Watch). *medRxiv* Published Online First: 2021. doi:10.1101/2021.03.22.21254130
11
Office for National Statistics. Coronavirus and vaccine hesitancy, Great Britain: 13 january to 7 february 2021. 2021.https://www.ons.gov.uk/peoplepopulationandcommunity/healthandsocialcare/healthandwellbeing/bulletins/coronavirusandvaccinehesitancygreatbritain/13januaryto7february2021
12

For peer review only

O'Dowd A. Covid-19: Black people over 80 in England are half as likely as white people to have been vaccinated. *BMJ* 2021;**372**. doi:10.1136/bmj.n357
13
Elgot J. Hancock: Most Bolton Covid patients eligible for jab but haven't had it. *Guardian* Published Online First: 16 May 2021.https://www.theguardian.com/world/2021/may/16/hancock-defends-india-travel-ban-delay-as-covid-variant-cases-mount
14
Schaffer DeRoo S, Pudalov NJ, Fu LY. Planning for a COVID-19 vaccination program. *JAMA* 2020;**323**:2458–9. doi:10.1001/jama.2020.8711
15
Mills E, Jadad AR, Ross C, *et al.* Systematic review of qualitative studies exploring parental beliefs and attitudes toward childhood vaccination identifies common barriers to vaccination. *Journal of Clinical Epidemiology* 2005;**58**:1081–8. doi:10.1016/j.jclinepi.2005.09.002
16
Quinn SC, Jamison AM, An J, *et al.* Measuring vaccine hesitancy, confidence, trust and flu vaccine uptake: Results of a national survey of White and African American adults. *Vaccine* 2019;**37**:1168–73.
17
Wilder-Smith AB, Qureshi K. Resurgence of measles in Europe: A systematic review on parental attitudes and beliefs of measles vaccine. *Journal of Epidemiology and Global Health* 2020;**10**:46–58. doi:10.2991/jegh.k.191117.001
18
Hornsey MJ, Lobera J, Díaz-Catalan C. Vaccine hesitancy is strongly associated with distrust of conventional medicine, and only weakly associated with trust in alternative medicine. *Social Science & Medicine* 2020;**255**. doi:10.1016/j.socscimed.2020.113019
19
Stoop N, Hirvonen K, Maystadt J-F. Institutional mistrust and child vaccination coverage in Africa. *BMJ Global Health* 2021;**6**. doi:10.1136/bmjgh-2020-004595
20
Momplaisir F, Haynes N, Nkwihoreze H, *et al.* Understanding drivers of Coronavirus Disease 2019 vaccine hesitancy among blacks. *Clinical Infectious Diseases* 2021;**73**. doi:10.1093/cid/ciab102
21
Kamal, A, Hodson, A, Pearce, JM. A rapid systematic review of factors influencing COVID-19 vaccination uptake in minority ethnic groups in the UK. *Vaccines* 2021;**9**. doi:10.3390/vaccines9101121
22
Razai MS, Osama R, McKechnie DGJ, *et al.* Covid-19 vaccine hesitancy among ethnic minority groups. *British Medical Journal* 2021;**372**. doi:10.1136/bmj.n513
23
Pinder RJ, Ferguson J, Møller H. Minority ethnicity patient satisfaction and experience: Results of the National Cancer Patient Experience Survey in England. *BMJ Open* 2016;**6**. doi:10.1136/bmjopen-2016-011938
24
Chauhan A, Walton M, Manias E, *et al.* The safety of health care for ethnic minority patients: A systematic review. *International Journal for Equity in Health*;**19**. doi:10.1186/s12939-020-01223-2
25

For peer review only

Allington D. Do institutional trust and experiences of healthcare explain demographic disparities in coronavirus vaccination uptake? 2021.https://osf.io/dfr3m
26
Wellcome. *Wellcome global monitor: How does the world feel about science and health?* London: : Wellcome Trust 2019.
27
Scottish Government. Inpatient experience survey. 2018.https://www.gov.scot/collections/inpatient-experience-survey/
28
Government U. Population of England and Wales. 2018.https://www.ethnicity-facts-figures.service.gov.uk/uk-population-by-ethnicity/national-and-regional-populations/population-of-england-and-wales/latest
29
R Core Team. *R: A language and environment for statistical computing*. Vienna, Austria: : R Foundation for Statistical Computing 2021. https://www.R-project.org/
30
Revelle W. *Psych: Procedures for psychological, psychometric, and personality research*. Evanston, Illinois: : Northwestern University 2021. https://CRAN.R-project.org/package=psych
31
Charles Dupont FEHJ with contributions from, others. many. *Hmisc: Harrell miscellaneous*. 2021. https://CRAN.R-project.org/package=Hmisc
32
Bischl B, Lang M, Bossek J, *et al. BBmisc: Miscellaneous helper functions for B. Bischl*. 2017. https://CRAN.R-project.org/package=BBmisc
33
Tingley D, Yamamoto T, Hirose K, *et al.* mediation: R package for Causal Mediation Analysis. *Journal of Statistical Software*;**59**:1–38. doi:10.18637/jss.v059.i05
34
Zhang Z, Mai Y. WebPower: Basic and advanced statistical power analysis. 2021. https://CRAN.R-project.org/package=WebPower

1 Linear models with standardised predictor and outcome variables.

For peer review only

STROBE Statement—Checklist of items that should be included in reports of ***cross-sectional studies***

	Item No	Recommendation	Page No
**Title and abstract**	1	(*a*) Indicate the study's design with a commonly used term in the title or the abstract	1,2
		(*b*) Provide in the abstract an informative and balanced summary of what was done and what was found	2-3
**Introduction**			
Background/rationale	2	Explain the scientific background and rationale for the investigation being reported	3-5
Objectives	3	State specific objectives, including any prespecified hypotheses	5
**Methods**			
Study design	4	Present key elements of study design early in the paper	5-6
Setting	5	Describe the setting, locations, and relevant dates, including periods of recruitment, exposure, follow-up, and data collection	6-7
Participants	6	(*a*) Give the eligibility criteria, and the sources and methods of selection of participants	6
Variables	7	Clearly define all outcomes, exposures, predictors, potential confounders, and effect modifiers. Give diagnostic criteria, if applicable	5-6
Data sources/ measurement	8*	For each variable of interest, give sources of data and details of methods of assessment (measurement). Describe comparability of assessment methods if there is more than one group	5-6
Bias	9	Describe any efforts to address potential sources of bias	6-7, 9
Study size	10	Explain how the study size was arrived at	6
Quantitative variables	11	Explain how quantitative variables were handled in the analyses. If applicable, describe which groupings were chosen and why	5-6
Statistical methods	12	(*a*) Describe all statistical methods, including those used to control for confounding	9
		(*b*) Describe any methods used to examine subgroups and interactions	N/A
		(*c*) Explain how missing data were addressed	6-7
		(*d*) If applicable, describe analytical methods taking account of sampling strategy	N/A
		(*e*) Describe any sensitivity analyses	9

For peer review only - http://bmjopen.bmj.com/site/about/guidelines.xhtml

**Results**			
Participants	13*	(a) Report numbers of individuals at each stage of study—eg numbers potentially eligible, examined for eligibility, confirmed eligible, included in the study, completing follow-up, and analysed	6, 8, 10
		(b) Give reasons for non-participation at each stage	N/A
		(c) Consider use of a flow diagram	N/A
Descriptive data	14*	(a) Give characteristics of study participants (eg demographic, clinical, social) and information on exposures and potential confounders	10
		(b) Indicate number of participants with missing data for each variable of interest	6-8
Outcome data	15*	Report numbers of outcome events or summary measures	10-11
Main results	16	(*a*) Give unadjusted estimates and, if applicable, confounder-adjusted estimates and their precision (eg, 95% confidence interval). Make clear which confounders were adjusted for and why they were included	13-16
		(*b*) Report category boundaries when continuous variables were categorized	6, 10
		(*c*) If relevant, consider translating estimates of relative risk into absolute risk for a meaningful time period	N/A
Other analyses	17	Report other analyses done—eg analyses of subgroups and interactions, and sensitivity analyses	9
**Discussion**			
Key results	18	Summarise key results with reference to study objectives	16
Limitations	19	Discuss limitations of the study, taking into account sources of potential bias or imprecision. Discuss both direction and magnitude of any potential bias	17
Interpretation	20	Give a cautious overall interpretation of results considering objectives, limitations, multiplicity of analyses, results from similar studies, and other relevant evidence	16-17
Generalisability	21	Discuss the generalisability (external validity) of the study results	17
**Other information**			
Funding	22	Give the source of funding and the role of the funders for the present study and, if applicable, for the original study on which the present article is based	18

*Give information separately for exposed and unexposed groups.

For peer review only

Note: An Explanation and Elaboration article discusses each checklist item and gives methodological background and published examples of transparent reporting. The STROBE checklist is best used in conjunction with this article (freely available on the Web sites of PLoS Medicine at http://www.plosmedicine.org/, Annals of Internal Medicine at http://www.annals.org/, and Epidemiology at http://www.epidem.com/). Information on the STROBE Initiative is available at www.strobe-statement.org.

For peer review only
